# Early impact on sleep and mental health during the mandatory social isolation of COVID-19 outbreak: an obser vational cross-sectional study carried out in Argentina

**DOI:** 10.5935/1984-0063.20200121

**Published:** 2022

**Authors:** Stella Maris Valiensi, Agustín L. Folgueira, Arturo Garay

**Affiliations:** 1 Hospital Italiano, Buenos Aires, Argentina, Neurology-Sleep Medicine - Ciudad de Buenos Aires - Please select an option below - Argentina.; 2 Centro de Educación Médica e Investigaciones Clínicas “Norberto Quirno” (CEMIC), Neurology- Sleep Medicine – Ciudad de Buenos Aires – Please select an option below – Argentina.

**Keywords:** REM Sleep Parasomnias, Coronavirus Infections, Anxiety Disorders, Depression, Sleep, Social Isolation

## Abstract

**Introduction:**

The mandatory social isolation (MSI) due to the pandemic caused by COVID-19 in the world produced many changes in sleep and different areas of mental health. Objectives: To evaluate the early effects of MSI on sleep, anxiety, and depression in Argentina.

**Material and Methods:**

An anonymous observational cross-sectional web-based study was distributed throughout the country and was completed by 2,594 respondents to analyze demographic information, quality of sleep, REM sleep-related events, depressive, and anxiety symptoms.

**Results:**

The study revealed that 53, 21, 22, 23, and 16% of people surveyed were poor sleepers, had dream-related behaviors, nightmares, depression, and anxiety symptoms, respectively. Multivariate logistic regression showed a positive correlation between anxiety, being a poor sleeper, and having nightmares.

**Conclusion:**

We identified the early effects of MSI on sleep quality, dreaming activity, anxiety, and depression in Argentina during the COVID-19 outbreak. Our findings can be used to formulate sleep and psychological interventions to improve mental health during the pandemic and post-pandemic times.

## INTRODUCTION

By the end 2019, a new coronavirus disease (COVID-19) appeared in Wuhan, China, and rapidly spread throughout the entire globe^1^. As a countermeasure to the COVID-19 outbreak, quarantine was imposed in several countries. Even though quarantine is a unique and exceptional measure used to control in this case, the spread of the COVID-19 pandemic, its implementation was associated with deleterious effects on public health, mental health, and economy^2^. In Argentina, the presentation of the first cases was observed in the first days of March. By March 20, with the increase of COVID-19 detected cases, the national government decided to install a rather strict quarantine including mandatory social isolation (MSI), closure of borders, cessation of activities except for those activities considered essentials. This period included the time of maximum restriction of the MSI (phase I) in our country from March 20^th^ to April 26^th^^3^.

It is very well-known that social relationships are central to human well-being and are critically involved in the maintenance of health, sleep, and mental health. Interestingly, some reports of people surveyed during the COVID-19 outbreak indicates an increase of dreaming mentation and disturbed dreaming. Indeed, experiences that essentially put “the life on hold”, as the COVID-19 outbreak, meaning this, separation from loved ones, lack of social interaction, confinement, loss of routine, uncertainty, and fear about the situation, economic injury, are directly related to the appearance of symptoms of insomnia, fatigue, excessive anxiety disorders, and irritability^4,5,6,7,8,9,10,11,12,13,14,15,16,17^.

In this observational/cross-sectional study, our purpose was to evaluate the early effects of MSI on sleep, anxiety, and depression symptoms in Argentina during the COVID-19 outbreak.

## MATERIAL AND METHODS

### Study design and participants

An anonymous, voluntary web-based observational cross-sectional survey was distributed through social media and email. The survey was enabled from April 13 to April 30. All subjects reported demographic and social data, COVID-19 related information, they completed questionnaires to evaluate the sleep and the presence of anxiety and depressive symptoms.

### Ethical statement

This study was conducted in accordance with the declaration of Helsinki. Before starting the questionnaire, the objectives of the study were explained. Participants could withdraw from the survey at any moment without providing any justification.

### Measurement tools

**Sleep quality:** The Spanish version of the PSQI (Pittsburgh sleep quality index) was used^18,19^. This is a 24 items scale that is divided into seven subcomponents (subjective sleep quality, sleep latency, sleep duration, habitual sleep efficiency, sleep disturbance, use of sleep medications, and daytime dysfunction). The score for each subcomponent ranges from 0 to 3 points. The global PSQI score ranges from 0 to 21, with higher scores indicating more severe sleep disorders. Scores of 5 or less were considered good sleepers while a score greater than 5 categorizes participants as bad sleepers^20^. A score of 5 to 7 was considered mild insomnia or mild sleep problem and requires the indication of medical treatment; a score of 8 to 14 implies moderate insomnia or moderate sleep problem that requires medical attention and treatment, and a score of 14 to 21 suggests a serious sleep problem^18,19,20,21^.

Additionally, we also asked about the nap habits during MSI: changes in frequency and/or duration of naps when compared with the period pre-COVID-19 outbreak.

**To assess the dream-related behaviors**^16^, we used a single question for REM sleep behavior Disorder (RBD1Q). Although there is some evidence showing poor performance of this tool for RBD evaluation^22,23^, it is validated in different languages for detecting REM sleep behavior disorders defined by a positive response and we considered is very appropriate to detect the presence of motor activity related to dreams due to its simplicity. The presence of nightmares or bad dreams was assessed by the PSQI question **#**H5 and was considered positive when these events occurred one or more times a week.

**To assess depression symptoms**, we used the patient health questionnaire – 9 (PHQ-9)^24,25,26^. The PHQ is a self-administered version of the PRIME-MD diagnostic instrument for common mental disorders. The PHQ-9 is the depression module, which scores each of the 9 DSM-IV criteria as “0” (not at all) to “3” (nearly every day). Scores range from 0 to 27 points. A PHQ-9 score ≥ of 10 has a sensitivity of 88% and a specificity of 88% for “major depression”. PHQ-9 scores of 5, 10, 15, and 20 represent mild, moderate, moderately severe, and severe depression, respectively.

**To detect anxiety symptoms**, we used the generalized anxiety detection questionnaire (GAD-7)^27^. The GAD includes 7 items on a 4-point Likert scale ranging from 0 (never) to 3 (nearly every day). The total score ranges from 0 to 21, with higher scores indicating more severe functional impairments as a result of anxiety. Scores of 5, 10, and 15 are taken as the cutoff points for mild, moderate, and severe anxiety, respectively^27,28^.

### Statistical analysis

The results of qualitative variables were expressed as frequencies and percentages. Quantitative variables, by means and standard deviation of the mean. Trend and distribution measurements were used to describe the groups. The t-test was used to analyze the continuous variables. Analysis of variance (ANOVA) was done using the R language. Chi-square tests were used to compare the qualitative variables. Binary logistic regression for multivariate analysis, between sex, age, bedtime, time to get up, sleep onset latency, hours of night sleep <6 hours and >10 hours, naps, nightmares, diagnosis of depression, anxiety, and poor sleepers. All data were analyzed for Statistical Package with Social Science (SPSS) version 18 (SPSS 18 Chicago, IL, U.S.A, SPSS Inc.). The *p*-values of less than 0.05 were considered statistically significant.

## RESULTS

We selected 2,594 participants from a total of 2,798 participants, which is 92.7% of the participants who answered the survey. Criteria for exclusion were: consecutively surveys that have equal entries were assumed to be duplicate responses and were therefore eliminated. Also, participants under 18 years of age, people who were living abroad at the time of the survey, erroneous or inconsistent data, sleep duration of fewer than 4 hours, and more than 12 hours, and naps duration greater than 3 hours were excluded.

### Demographic characteristics

The demographic characteristics of participants are shown in Table 1. There were 2,594 participants in our study, 803 participants being males (31%), 1,790 females (69%) and 1 (0.04%) non-binary.

**Table 1.** Demographic characteristics of the sample. Scores outcomes over a total of N=2,594 subjects.

	Mean	SD
Age (18-85 years old)	42	13
COVID-19 Time exposition on healthcare workers (hours)	7	12.4
	**n**	**%**
AMBA (Buenos Aires city and surrounding Municipalities)	745	28.7
Buenos Aires province (outside AMBA)	984	37.9
Provinces (outside Buenos Aires province)	865	33.3
**Gender**		
Male	803	31
Female	1,790	69
Non binary	1	0.04
**Age**		
18 to 40 years old	1,243	47.9
40 to 55 years old	884	34.1
55 to 65 years old	326	12.6
65 to 85 years old	141	5.4
**Employed**		
Healthcare workers	710	27.4
Employed	1,040	40.1
Unemployed	844	32.5

The mean age of the participants was 42.0 (SD: ±13) years. The most represented age range was 40-55 years (52.1%). Regarding their employment situation, 1,750 participants had stable jobs (27.4% were health workers) and 844 participants (32.5%) were unemployed.

In our study, most of the people live in AMBA (Buenos Aires city and surrounding municipalities): 745 (28.7%) and Buenos Aires province (outside AMBA) 984 (37.9%), while the rest, 865 (33.3%) were distributed across 14 provinces (we did not receive data from one of them, Jujuy province).

### Survey general description

Table 2 shows the characteristics of the overall sample related to sleep, naps, nightmares, dream-enacted behaviors by PSQI, RBDQ1 questions. The mean bedtime was 00:02 hours, the mean of sleep onset latency was prolonged (37.8min), and wake up time was at 8:29 a.m. The mean nap duration was 68 min. (range: 5-180min.). Naps were reported to be more frequent in 516 surveys (20%) and of longer duration in 756 surveys (29%). The subjective sleep efficiency was 83.5% (range: 40-100).

**Table 2.** Full sample of sleep and naps questions. Scores outcomes over a total of N=2,594 subjects.

	Min	Max	Mean	SD
**Bedtime** (hh:min)	20:00	04:00	**00:02**	1:22
**Get up** (hh:min)	04:00	14:00	**08:29**	1:38
**Sleep duration** (hh:min)	04:00	12:00	**07:00**	1:18
**Time in bed** (Time Down – Time Up) (hh:min)	04:00	14:00	**08:26**	1:25
**Sleep onset latency** (min)	0	240	**37.82**	40.35
**Latency time to get up** (min)	0	240	**23.06**	24.01
**Sleep efficiency** (%)	40	100	**83.5**	13.18
**Nap duration time** (min)	5	180	**67.99**	36.3

In relation before MSI							
	**n**	**%**		**n**	**%**	**n**	**%**
Bedtime: no changes	1083	41.8	Naps Frequency: not nap	1114	42.9 Naps Duration: not nap	1114	42.9
Bedtime: earlier	826	31.8	Naps Frequency: no changes	753	29 Naps Duration: no changes	516	19.9
Bedtime: later	685	26.4	Naps Frequency: decreased	360	13.9 Naps Duration: shorter	630	24.3
			Naps Frequency: increased	367	14.1 Naps Duration: longer	334	12.9

Table 3 shows the score outcomes for PSQI. The results shows that 47% of answers reported an increase of sleep onset latency (>30min ≥1 night in the week). Altered sleep continuity was observed by the following: getting up at night to go to the bathroom: 1,336 answers (51.5%); having pain: 695 answers (27%); feeling too hot: 665 answers (26%), feeling too cold: 630 answers (24%). The global PSQI score of 5 or less, that were considered good sleepers corresponded to 1,219 answers (47%).

**Table 3.** Full sample of PSQI. Scores outcomes over a total of N=2,594 subjects.

	Mean	SD
PSQI#1 Bed Time (hh:mm)	00:02	1:22
PSQI#2 Sleep Onset Latency (minutes)	37.82	40.4
PSQI#3 Wake Up Time (hh:mm)	08:29	1:38
PSQI#4 Sleep duration time (hh:mm)	07:00	1:18
	**n**	**%**
PSQI#5a Sleep latency >30min ≥ 1/7 days	1,220	47
PSQI #5b Wake up in the night ≥ 1/7 days	1,514	58.4
PSQI #5c Getting up at night to go to the bathroom ≥ 1/7 days	1,336	51.5
PSQI#5d Not being able to breathe well ≥ 1/7 days	219	8.4
PSQI #5e To cough or to snore loudly≥ 1/7 days	400	15.4
PSQI #5f To feel too cold ≥ 1/7 days	630	24.3
PSQI #5g To feel too hot≥ 1/7 days	665	25.6
PSQI #5h To have nightmares or bad dreams ≥ 1/7 days	593	22.9
PSQI #5i To have pain ≥ 1/7 days	695	26.8
PSQI #5j Other reason(s) ≥ 1/7 days	466	18
PSQI # 6 Sleep quality overall fairly or very bad	891	34.3
PSQI #7 Take medicine to sleep ≥ 1/7 days	357	13.8
PSQI #8 Drowsiness while driving, eating, or doing other activities ≥ 1/7 days	222	8.6
PSQI #9 Encouragement to carry out activities of daily living ≥ 1/7 days	511	19.7
PSQI score ≤ 5: Good sleepers	1,219	**47**
PSQI score > 5: Poor sleepers	1,375	**53**
**Dreams-related behaviors: RBDQ1**	549	21.2

The question about nightmares or bad dreams (PSQI#5h), was answered positively by 22.9% of the people surveyed. For the evaluation of the presence of dream-related behaviors, we used the RBDQ1. We found that 549 (21.2%) of the people surveyed responded positively to this question.

Table 4 shows the analysis of scores of PSQI (sleep quality), PHQ-9 (depression), and GAD-7 (anxiety). The results revealed that 1,375 surveys (53%) were poor sleepers and 31.1% had mild insomnia, 32,4% had moderate insomnia, and 2.1% had a serious sleep problem. 21.1% of the people showed elevated values of depression symptoms by PHQ-9 (PHQ-9≥10) and 16% showed elevated levels of anxiety by GAD-7 (GAD-7 score ≥10).

**Table 4.** Analysis of the scores according severity of PSQI (sleep quality), PHQ-9 (depression), and GAD-7 (anxiety). Scores outcomes over a total of N=2,594 subjects.

PSQI score	n	%	PHQ-9 score	n	%	GAD-7 score	n	%
0-≤5: no problem	892	34.4	0-4: negative	1007	38.8	0-4: negative	1161	44.8
5-7: mild insomnia	807	31.1	5-9: mild	1039	40.0	5-9: mild	1114	42.9
8-14: moderate insomnia	842	32.4	10-14: moderate	384	14.8	10-14: moderate	234	9.0
14-21: severe insomnia	54	2.1	15-19: moderately severe	129	5.0	15-21: severe	85	3.3
			20-27: severe	35	1.3	10-21: anxiety	416	16.0
			10-27: depression	548	21.1			

Table 5 shows the relationship between the depression (PHQ-9), anxiety (GAD-7), and sleep quality (PSQI) questionnaires using univariate ANOVA (analysis post hoc Turkey test). The results indicate that there is a statistically significant positive correlation between the increase of the scores for depression and anxiety and the PSQI. The difference was significant among all PHQ-9 scores and the difference was significant between all GAD-7 severity grades (except between moderate and severe).

**Table 5.** Relationship between the depression (PHQ-9), anxiety (GAD-7) and sleep quality (PSQI) questionnaires using ANOVA univariate. Analysis post hoc Tukey Test. Scores outcomes over a total of N=2,594 subjects.

	n	Mean PSQI	SD	p
**Score PHQ-9**				
0-4 negative	1007	4.47	2.61	
5-9 mild	1039	6.80	3.04	
10-14 moderate	384	8.41	3.39	0.000
15-19 moderately serious	129	9.79	3.70	
20-27 severe	35	11.83	3.17	
**Score GAD-7**				
0-4: minimal	1161	4,84	2.81	
5-10: mild	1114	7.18	3.33	
11-15: moderate	234	8.79	3.33	0.000
16-21: severe	85	9.42	3.88	

Notes: *The difference was significant among all PHQ-9 scores; **The difference was significant between all GAD-7 severity grades (except between moderate and severe).

Table 6 shows multivariate analysis of binary logistic regression of different questionnaires over a total of n=2,594 subjects. Taking into account the effect of sex, age, bedtime greater than 00:00 hour, time to get up greater than 08:00 hour, sleep onset latency greater than 30 minutes, sleep duration less than 6 hours, sleep duration greater than 10 hours, take naps, the presence of nightmares, diagnosis of depression, anxiety and poor sleepers, we found as an important correlation that the latency to sleep greater than 30 minutes, the risk of being a poor sleeper was 3.51 and 3.69 the risk, for sleeping less than 6 hours. Having depression (PHQ-9≥10) the risk was 2.99, and having anxiety (GAD≥10) the risk of being a bad sleeper was 1.78. When analyzing nightmares, the risk of having them was 2.02 higher, in those with dream enacting behaviors by positive RBDQ1.

**Table 6.** Multivariate analysis of binary logistic regression of different questionnaires over a total of N=2,594 subjects.

	PSQI			RBDQ1			PHQ-9			GAD-7		
	OR	IC95	p	OR	IC95	p	OR	IC95	p	OR	IC95	p
**Gender female**	1.28	1.1-1.6	0.01	1.50	1.2-1.9	0.00	1.28	1.1-1.6	0.01	1.50	1.2-1	0.00
**Age <55 years old**	0.86	0.7-1.1	0.19	1.76	1.4-2.4	0.00	2.13	1.5-3	0.00	2.47	1.6-3.8	0.00
**Age ≥65 years old**	1.23	0.8-1.8	0.29	1.74	1.1-2.9	0.03	1.23	0.8-1.8	0.29	1.69	1.1-2.6	0.01
**Bedtime >12:00 p.m.**	0.79	0.7-0.9	0.02	1.05	0.9-1.3	0.65	1.28	1.0-1.6	0.04	1.17	0.9-1.5	0.27
**Wake up time >08:00 a.m.**	1.46	1.2-1.8	0.00	1.02	0.8-1.3	0.90	1.06	0.8-1.4	0.66	0.74	0.6-1	0.04
**Sleep onset latency >30min**	3.51	2.9-4.2	0.00	1.00	0.8-1.2	0.99	1.19	0.9-1.5	0.15	1.21	0.9-1.6	0.15
**Short sleeper (<6hs)**	3.69	2.7-5.1	0.00	1.02	0.8-1.4	0.89	1.54	1.1-2.1	0.01	1.73	1.2-2.4	0.00
**Long sleeper (>10hs)**	0.70	0.4-1.2	0.18	1.06	0.0-1.9	0.83	2.12	1.2-3.8	0.01	0.54	0.3-1.2	0.13
**Take naps**	1.19	0.9-1.4	0.05	1.18	0.3-1.4	0.22	1.26	1-1.6	0.04	1.00	0.8-1.3	1.00
**Cough or snore (≥1/7 days)**	1.75	1.4-2.3	0.00	1.88	1.5-2.4	0.00	0.97	0.7-1.3	0.83	1.25	0.9-1.7	0.18
**Nightmares (≥1/7 days)**	1.83	1.5-2.3	0.00	2.02	1.6-2.5	0.00	1.50	1.2-1.9	0.00	2.12	1.6-2.8	0.00
**PSQI>5**				1.15	0.9-1.4	0.21	2.97	2.3-3.9	0.00	1.81	1.4-2.4	0.00
**PHQ-9≥10**	2.99	2.3-3.9	0.00	1.06	0.8-1.4	0.68				7.52	5.8-9.7	0.00
**GAD≥10**	1.78	1.3-2.4	0.00	1.37	1.0-1.8	0.02	7.51	5.8-9.7	0.00			
Cox y Snell coefficient		22.0			4.6			21.4			19.2	
Nagelkerke coefficient		29.4			7.4			33.3			32.9	

Taking into account the same variables analyzed, we found that being under 55 years of age the risk was 2.13 higher in those who presented depression, as well as those who slept more than 10 hours, the estimated risk was 2.47, in those who had anxiety. The risk of having depression was 7.4 times higher, and being a poor sleeper the risk was 2.4 times higher than good sleepers.

Taking into account the same variables, we found that having depression, the risk was 7.51 times higher in those who presented anxiety and being a bad sleeper the risk was 2.99 in those who were anxious.

## DISCUSSION

Our study attempts to give a first look at early manifestations of sleep alterations from a multidimensional perspective about symptoms of insomnia, nightmares, dream-related behaviors and depression and anxiety symptoms during the first month of MSI in Argentina.

One of the first findings was that more than half of the population was poor sleepers reporting in their surveys the following: prolonged sleep onset latency, prolonged time to bedtime and late get up, an increase of the number and time duration of sleep naps and in the number of awakenings mainly produced by cough access, snore or nightmares. This population of poor sleepers was correlated with higher levels of anxiety and depression during MSI. Besides, almost half of the population reported not being able to fall asleep during the first hour, pointing out a probable phase delay. This in agreement with the well-known observation that the effect of physical distancing and the excessive use of ALAN (Artificial Avant Light) could be related to an increased risk of circadian rhythm dysregulation^30,31^.

In pre-COVID-19 times, we estimated that about 20% of the general population of Argentina sleep poorly, a percentage that could be increased up to 50% or more in some specific groups (drivers, adolescents, population with a low socioeconomic level)^32^. In the present study, we found that the total subjective sleep time was within the values considered normal for adults^33^ and that the disturbance of the sleep continuity was the hallmark of this issue. To compare the prevalence of sleep quality before the MSI, in the general population, we found that, to our knowledge, there were no studies in Argentina reporting this condition by PSQI. In our study, we observed a huge impact on sleep quality, i.e., we found that 64.3% of the population had insomnia symptoms ranging from mild to severe insomnia symptoms during approximately the first month of MSI. To compare the prevalence of insomnia to our knowledge, there were no studies in Argentina reporting this condition before the MSI. Interestingly, research carried out in the general population of Latin America, including metropolitan areas of Ciudad de México (México), Montevideo (Uruguay), Santiago de Chile (Chile), and Caracas (Venezuela), reported a prevalence of insomnia of about 34.7% (33.3% to 36%)^34^.

As for napping, 20% of people surveyed reported increasing the frequency of naps and a third sleeping more minutes than before MSI. Napping, in this study, was correlated with being bad sleepers and presenting nightmares. We did not find any previous studies that analyzed these data in Argentina.

Some informal publications and at present several scientific works reported an increase of dreaming and disturbed dreaming during the COVID-19 outbreak^44,45,46,47^. Our results show that more than 20% of the people surveyed responded positively about presenting dream-related behaviors by question RBDQ1. This positive answer was mostly correlated with individuals under the age of 55 years old and with those who manifested fragmenting their sleep by coughing, snoring, or nightmares. It was also positively correlated with having anxiety disorder according to GAD-7. As far we know, only a single study, performed three months after MSI, analyzed the presence of dream-enacting behaviors in health workers, with similar results^36^. These findings are in agreement with concepts expressed by Nielsen et al. (2019)^16^ in the normal population. In our sample, they could be related to the emotional impact denoted by increased anxiety and depression symptoms during MSI stressful conditions. The necessity of characterizing the phenomenology of dream enacting behaviors had been pointed out by two recent and excellent reviews^64,65,66^.

The presence of nightmares was observed in 23% of the people surveyed. The presence of nightmares was correlated with being poorly sleeper, acting dreams by RBDQ1, having depressive symptoms, and anxiety. Nightmares are commonly defined as disturbing dreams characterized by awakening from (REM) sleep with very vivid and detailed dream recall. Dream content is typically related to threats to survival, security, or self-esteem^36^. For the diagnosis of nightmare disorder, dream content and the awakening must cause clinically significant distress or impairment in important areas of functioning^37^. At least one nightmare per week is often used as a criterion of clinical significant distress^38^. Anxiety or fear is often reported as the predominant emotions of nightmares; however, recent research suggests that other, less intense emotions like frustration or guilt can be involved as well^39^. The prevalence of frequent nightmares in the general population is about 5% in adults^40,41^. The theories about the origin of nightmares/bad dreams^42^ are multifaceted: mechanisms for regulating emotions, mastering stress, de-somatization of affection, contextualization of the emotional, and extinction of fear memory. A failure in the mechanisms used by nightmares would predispose to their perpetuation and the possibility of developing the disease. This is very commonly observed in patients with posttraumatic stress after a few months or years after the life event and can last for several years.

In times of COVID-19 pandemic, recent publications show that the significant increase in dream recall might be explained by three factors: first, for many people, their sleep patterns have changed dramatically during the pandemic. This might be especially true among young adults, who are sleeping longer, and thus, they were able to recall more^43^. Second, the combination of more negatively toned dreams and more dreams relating to the pandemic should result – according to the salience hypothesis of dream recall in higher dream recall^45^. Lastly, one of the symptoms of insomnia is the more frequent nocturnal awakenings, which has been found to correlate with increased dream recall; thus, the increase in insomnia prevalence due to the pandemic^7,9^ might have also increased dream recall.

Early this year, when stay-at-home directives were put in place widely, society quite unexpectedly experienced what it was called a dream surge: a global increase in the reporting of vivid, bizarre dreams, many of which are concerned with coronavirus and social distancing. By early April, social and mainstream media outlets had begun broadcasting the message: the world is dreaming about COVID-19. The association between nightmares and stressful situations is widely known^45,46^. Gupta (2020)^17^ reported nightmares or disturbing dreams or nightmares in patients with posttraumatic stress disorders (PTSD), and Nielsen et al. (2020)^47^reported that this increase was attributed by several authors^48,49^ to the economic and social consequences produced by the COVID-19 outbreak. They found that less than one-third of the population presented these REM sleep-related events. So far, we did not find the percentage of these alterations in the world population during pandemic times in the current literature, which makes our findings interesting.

Depression is a frequent psychiatric illness characterized by depressive mood, loss of interest or pleasure, feelings of guilt, disability, sleep and appetite disturbances, lack of energy, and difficulties in concentration and there are several causes for these symptoms^50,51,52,53^. We noticed that it was associated with being younger than 55 years old, presenting anxiety disorders, sleeping more than 10 hours, and being bad sleepers. The anxiety is correlated with poor sleepers^54,55^. For this reason, we believe that reinforcing the action of external zeitgebers, such as encouraging physical activity^56,57^, slowly restoring social rhythms, promoting exposure to light, maintaining regular sleeping and feeding times^58,59^, would help to improve mood changes. With this observation, the risk of PTSD, already identified in previous studies, emerges as an imminent problem to consider^60,61^. Finally, the arrival of the first cases of COVID-19 occurred months later than in China and Europe. During the first months of 2020, Argentinian people were viewers of the real consequences of the outbreak in the first world countries, hence, a state of alert and anxiety about an inevitable outcome could have been generated prematurely.

The impact observed with the scales used and its consistency with previous reports related to COVID-19/SARS/MERS/Ebola outbreaks points to the role of the state of alert and anxiety activating the hypothalamus-pituitary-adrenal (HPA) system could promote a vicious cycle of stress and insomnia, and their consequent effects on the score of anxiety and depression^62^. A study conducted one week after the onset of MSI showed depressive and anxiety symptoms of 23% and 33%, respectively^63^. The present study evidenced similar depression but less percentage in anxiety one month after the onset of MSI. Perhaps by “handling” it, through nightmares and behavioral changes of REM sleep as we have seen at approximately one month of the start of the MSI (see Table 5). Although many people tend to react resiliently to stress, others appear to display depression-related symptoms, anxiety, and sleep disorders.

The biases of this study were various, for example, it was an online self-report questionnaire which leads to a sample bias, one of this was that 69% of people surveyed were women, which should lead us to take the results of differences by gender with caution and also, we did not ask about alcohol or drugs consumption as sleep inducers, weight gain, cognitive impairment or hours to exposure to light.

Concluding, we identified for the first time, early effects of MSI on sleep quality, dream enacting behaviors, nightmares, depression, and anxiety symptoms in Argentina during the COVID-19 outbreak. Our findings can be used to formulate sleep and psychological interventions trying to improve mental health during the pandemic and post-pandemic times.
